# Evaluation of persuasiveness of messages to reduce stigma towards bulimia nervosa

**DOI:** 10.1186/2050-2974-2-S1-O41

**Published:** 2014-11-24

**Authors:** Siân A McLean, Susan J Paxton, Jonathan M Mond, Phillipa J Hay, Bryan Rodgers

**Affiliations:** 1 School of Psychological Science, La Trobe University, Melbourne, Australia; 2 Department of Psychology, Macquarie University, Sydney, Australia; 3 School of Medicine, University of Western Sydney, Sydney, Australia; 4 School of Sociology, Australian National University, Canberra, Australia

## 

Public health interventions are needed to reduce stigma towards bulimia nervosa (BN) to enhance appropriate treatment seeking. This study aimed to evaluate persuasiveness of health messages designed to increase mental health literacy about BN. A community sample of adults (N = 2092) completed self-report measures of knowledge about BN, stigma towards BN and ratings of persuasiveness of health messages on dimensions of convincingness and likelihood of changing attitudes. Messages highlighting that BN is a serious mental illness and that BN has nothing to do with attention seeking were rated as significantly more convincing and significantly more likely to change one's and others' attitudes towards BN than messages describing treatment options for BN. Ratings of message convincingness were positively associated with knowledge about BN for males, but not for females. Knowledge was not related to likelihood of changing attitudes. Message convincingness was inversely related to dimensions of stigma, including perceived advantages of BN, and perceptions that people with BN are unreliable and personally responsible for their illness. No relationship was found between stigma and likelihood of messages changing attitudes. The findings from this study provide a clear direction for implementation in public health interventions of particular messages to reduce BN stigma.

This abstract was presented in the **Prevention & Public Health** stream of the 2014 ANZAED Conference.

